# The impact of COVID-19 on intestinal flora: A protocol for systematic review and meta analysis

**DOI:** 10.1097/MD.0000000000022273

**Published:** 2020-09-25

**Authors:** Fangyuan Li, Hua Lu, Xinyun Li, Xinxin Wang, Qi Zhang, Ling Mi

**Affiliations:** a College of Clinical Medicine, Chengdu University of Traditional Chinese Medicine; b Chengdu University of Traditional Chinese Medicine Affiliated Hospital; c College of Acupuncture and Tuina, Chengdu University of Traditional Chinese Medicine; d Maternal and Child Reproductive Hospital Affiliated to Chengdu University of Traditional Chinese Medicine, Chengdu, Sichuan Province, P.R. China.

**Keywords:** coronavirus disease 2019, intestinal flora, novel coronavirus, systematic review

## Abstract

**Background:**

Coronavirus disease (COVID-19) sparked global concern for its outbreak and pandemic. It caused severe respiratory tract infections and a significant proportion of patients with gastrointestinal symptoms. Several studies have investigated the intestinal flora of COVID-19. However, so far there has been no evidence demonstrating the evidence on the association of COVID-19 with intestinal flora through meta-analysis. A systematic and comprehensive understanding of their relationship is essential to provide public health prevention or treatment strategy.

**Methods and analysis:**

This systematic review and meta-analysis will be reported following the Preferred Reporting Items for Systematic Reviews and Meta-Analyses guidelines. Observational studies (cohort studies, case-control, and cross-sectional studies) and clinical trials will be eligible. Studies eligible for inclusion must contain participants with COVID-19. Systematic searches will be conducted in PubMed, EMBASE, Cochrane Library, Ovid, EBSCO, World Health Organization COVID-19 database, China National Knowledge Internet, WanFang Data, Chinese Scientific and Technological Journal Database, and Chinese Biomedical Databases. A pre-designed search strategy of medical subject headings and free text terms for COVID-19 and intestinal flora will be used. Two reviewers will independently screen the titles and abstracts, followed by full-text screening. Discrepancies will be resolved by consensus with a third reviewer. The reviewers will then extract data from each eligible article based on PECOS (Population, Exposure, Comparator, Outcomes, and Study design). The risk of bias and quality of included studies will be assessed using an appropriate tool. A random-effects meta-analysis will be considered where there are sufficiently homogeneous studies; otherwise, a narrative synthesis will be conducted. Heterogeneity among studies will be assessed using I^2^ statistics. If substantial heterogeneity detected, subgroup analyses and meta-regression will be conducted to look for the potential causes.

**Ethics and dissemination:**

Ethical approval is not required as we will use data from published articles. Findings will be published in a peer-reviewed journal.

PROSPERO registration number: CRD42020191640

## Abstract 2

Supplemental Digital Content is available in the text

## 1 Introduction

Since novel coronavirus was first discovered in December 2019, an extremely high potential for dissemination resulted in the global coronavirus disease 2019 (COVID-19) pandemic in 2020. The mortality rate of COVID-19 is much higher than that of any common influenza, affecting millions of people worldwide. As of July 25, 2020, 15,581,009 confirmed cases of COVID-19, including 635,173 deaths, were reported to World Health Organization.^[1]^ COVID-19 is caused by a beta coronavirus called SARS-CoV-2 and similar to severe acute respiratory syndrome and Middle East Respiratory Syndrome, which affects the lower respiratory tract and manifests as pneumonia in humans.^[2]^ The most common symptoms were fever, cough, expectoration, haemoptysis, headache, myalgia, diarrhea, and fatigue.^[3]^ A significant proportion of these patients had gastrointestinal (GI) symptoms.^[4]^ Some researches indicated that SARS-CoV-2 might be spread by fecal-oral transmission, and diarrhea could be a presenting feature in the incubation period.^[5,6]^ Unfortunately, there are no drugs or vaccines effective for the prevention or treatment of COVID-19 patients in large-scale studies.^[7,8]^ As a public health emergency of international concern, global disease control of COVID-19 is challenging.

The gastrointestinal tract and respiratory tract are part of a shared mucosal immune system termed the gut-lung axis.^[9]^ An increasing amount of evidence supports the existence of the gut-lung axis.^[10]^ The gastrointestinal tract and the respiratory tract's microbiota participate in the gut-lung axis, influencing both local, and distant immune responses.^[9]^ The intestinal microbiota supports local mucosal immunity and is increasingly being recognized as an essential modulator of the systemic immune system.^[11]^ Accumulating evidence indicates that intestinal flora influences lung immunity.^[12]^ However, it is essential to note that the gut-lung axis is bidirectional and is one means of communication between the gut microbiota and the lungs.^[13]^ Respiratory diseases have long been associated with lung-gut axis.^[13,14]^ Recent findings now highlighted the essential roles for gut microbiota in shaping lung inflammation.^[15]^ Interestingly, there is a bidirectional interaction between respiratory tract infections and gut microbiome. The mutual interactions mostly focused on asthma, acute, and chronic respiratory infections, have received increasing attention.^[16–19]^ Influenza virus infection is believed to cause changes in intestinal flora.^[20]^ A previous study demonstrated that the composition and diversity of gut microbiota were altered after viral lung infections. For instance, viral lung infection resulted in an increase in the phylum *Bacteroidetes* and a corresponding decrease in the *Firmicutes phylum*.^[20]^ While the gut microbiome also shapes the adaptive immune responses against respiratory pathogens.^[21]^ Intestinal flora is closely related to respiratory virus infection and may influence the occurrence and development of diseases through the gut-lung axis.^[18]^ Thus, the gut Microbiota may play a potential role in the treatment of lung diseases.

Novel coronavirus also has an impact on the intestinal flora. Compared with healthy controls, COVID-19 patients had significantly reduced bacterial diversity, a considerably higher relative abundance of opportunistic pathogens.^[18]^ The gut microbiome of the COVID-19 group was dominated by *Streptococcus*, *Rothia*, *Veillonella*, *Erysipelatoclostridium*, and *Actinomyces*, whereas the microbiome of health group was dominated by the genera *Romboutsia*, *Faecalibacterium*, *Fusicatenibacter*, and *Eubacterium hallii* group.^[23]^ Concurrently, patients with COVID-19 were characterized by enrichment of opportunistic pathogens and depletion of beneficial commensals.^[22]^ The baseline abundance of *Clostridium ramosum*, *Coprobacillus*, and Clostridium hathewayi correlated with COVID-19 severity, while the abundance of Faecalibacterium *prausnitzii* (an anti-inflammatory bacterium) was negatively correlated with disease severity.^[22]^*Bacteroides thetaiotaomicron*, *Bacteroides massiliensis*, *Bacteroides dorei*, and *Bacteroides ovatus* correlated inversely with SARS-CoV-2 load in fecal samples from patients throughout hospitalization.^[22]^ Indeed, accumulating evidence indicates that microbiota can modulate the immune response in the course of both bacterial and viral infections, becoming a potential target in the management of all these diseases.^[23]^ The recent pandemic induced by COVID-19 reminded us that the potential value of the gut microbiota may be as a therapeutic target for COVID-19. It may be possible to look in the gut for a solution or mitigation of SARS-CoV-2 infection.^[24]^

As of yet, there has been no systematic review and meta-analysis of studies reporting on the relationship between COVID-19 and intestinal flora. A better understanding of the relationship between gut microbiota and COVID-19 to derive appropriate targets for prevention or treatment is needed. Therefore, we aim to ascertain the association between COVID-19 and intestinal flora that will facilitate management or prevention strategies of COVID-19.

## 2 Methods and analysis

This protocol is registered in the International Prospective Register of Systematic Reviews (PROSPERO), registration number CRD42020191640. We designed this systematic review and meta-analysis following the Preferred Reporting Items for Systematic Reviews and Meta-Analyses Protocol statement^[25]^ and meta-analysis of Observational Studies in Epidemiology.^[26]^

### 2.1 Inclusion/exclusion criteria for study selection

#### 2.1.1 Study designs and characteristics

Studies concerning the association between COVID-19 and intestinal flora, which meet all inclusion criteria, will be included in the systematic review. We will include cohort studies (both prospective and retrospective cohort studies), case-control studies, cross-sectional studies, and clinical trials. Report of the studies in any language is eligible to be included. Reviews, commentaries, short surveys, case reports, and letters will be excluded. Review inclusion criteria are specified according to Participant, Intervention (or Exposure), Comparator and Outcome.

#### 2.1.2 Participants

Participants with COVID-19; not have any severe primary GI disease that can affect intestinal flora or taking probiotics for a long time that affect intestinal flora; could be received anti-viral therapy.

#### 2.1.3 Exposure

The exposures of interest are infection with COVID-19 (determined from throat swabs).

#### 2.1.4 Comparators

The comparator will be healthy population that without COVID-19.

#### 2.1.5 Outcome measures

The outcome will be the changes of gut microbiota, which explicitly reported at least 1 of the following: fecal mycobiome profiles, the composition of intestinal flora, changes in the fecal fungal, or bacterial microbiomes, the abundance of opportunistic pathogens, the abundance of beneficial commensal bacteria, and gut microbiota diversity.

#### 2.1.6 Information sources and search strategy

The search strategies were performed by FYL and XXW, and differences were resolved by discussion with a third reviewer (XYL). We conducted searches in PubMed, EMBASE, Cochrane Library, Ovid, EBSCO, World Health Organization COVID-19 database, China National Knowledge Internet, WanFang Data, Chinese Scientific, and Technological Journal Database, and Chinese Biomedical Databases. These databases will be searched for relevant articles, from November 2019 until 2021/04/30.

**Figure 1:**
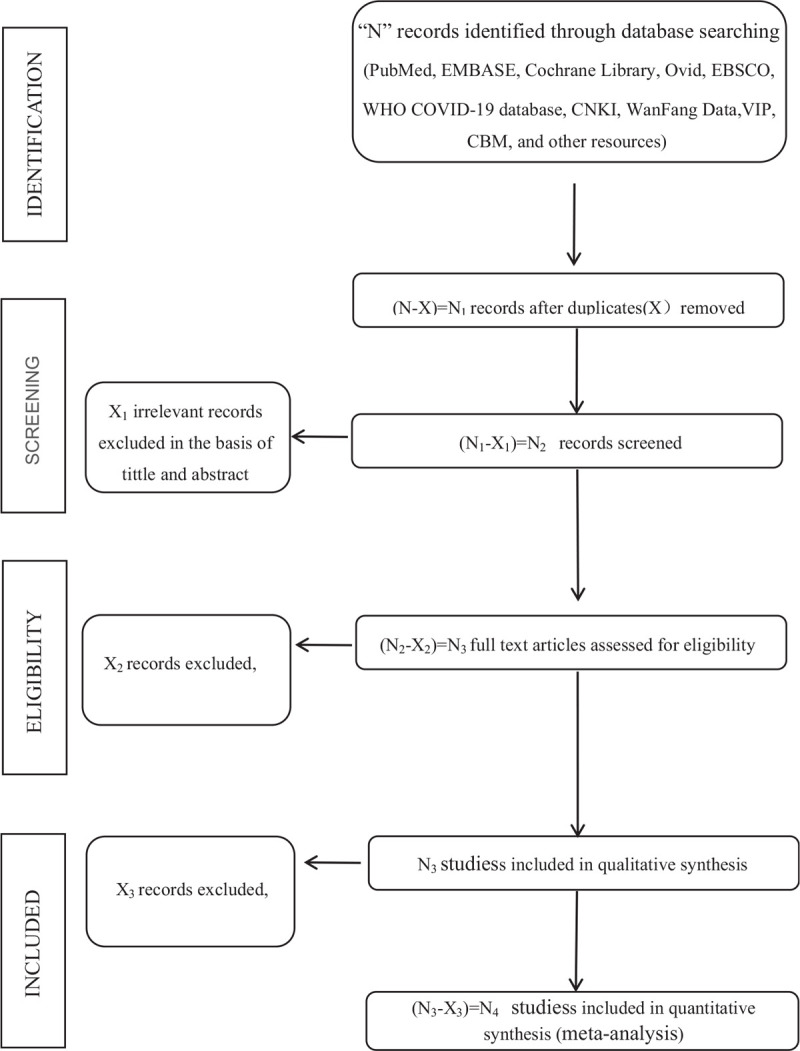
flowchart.

The search will consist of searching medical subject heading(MeSH) terms and free text (in the title and abstract) for the concepts “COVID-19” and “intestinal flora” (combined with the Boolean logic operation “AND”). The following search strategy will be used for PubMed (see online supplementary appendix 1), which will then be adapted for other databases to be searched. At the same time, we will search for the clinical trial registries (i.e., https://clinicaltrials.gov/) and gray literature about coronavirus infections and intestinal flora on the corresponding website to complete the electronic databases’ deficiencies. Conference proceedings and academic exchange summaries will be manually retrieved. To identify other relevant study data, we will contact the first author or correspondent author via email or telephone to obtain incomplete data. The whole process of study selection is summarized as flowchart in Figure 1.

### 2.2 Study selection

Articles from the database searches will be imported into Endnote X9 software. Duplicates will be removed. Two reviewers (XXW and QZ) will independently screen titles and abstracts in parallel to select studies for inclusion. Records that meet the specified inclusion criteria will then be taken forward to full-text screening, and records with little information available will be excluded. Potentially eligible full-text reports will be scrutinized carefully to decide whether to include or not. The reviewers will repeatedly cross-check the results and indicate the reasons for rejecting the article. Disagreement in the above process will be solved through discussion with a third reviewer (XYL) where necessary.

### 2.3 Data extraction

Data extraction will be performed by 2 authors independently (FYL and QZ). Any disagreements will be settled through discussion or the involvement of a third reviewer (XYL). Data from each eligible article will be extracted and compiled using a standardized Excel spreadsheet. Eligible extracted items will be obtained following the PECOS steps (Population, Exposure, Comparator, Outcomes, and Study design. The following data items will be extracted:

#### 2.3.1 Population

Participants’ demographic factors (e.g., mean age, ethnic distribution, proportion of gender, body mass index), inclusion and exclusion criteria, comorbidities (e.g., GI disease), medication intake.

#### 2.3.2 Exposure

Diagnosis of COVID-19, number of exposed subjects, details of COVID-19 severity.

#### 2.3.3 Comparators

Number of unexposed subjects.

#### 2.3.4 Outcomes

Identification of intestinal flora outcomes (intestinal flora measurement method), changes in the gut microbiota (i.e., fecal mycobiome profiles, the composition of intestinal flora, changes in the fecal fungal, or bacterial microbiomes, the abundance of opportunistic pathogens, the abundance of beneficial commensal bacteria, and gut microbiota diversity), any association between COVID-19 and intestinal flora, any risk estimate between COVID-19, and changes in the intestinal flora.

#### 2.3.5 Study characteristics

Title, objective, study design, country/region, journal, first author, sample size, observation, or follow-up time time.

Once the data is extracted, the above information should be cross-checked by 2 reviewers independently.

### 2.4 Quality and bias assessment

The methodological quality of the included studies will be assessed by 2 reviewers (FYL and XYL) using the risk of bias (ROB). Two independent reviewers will be blinded to the titles, authors, and years of publication of the studies to evaluate the ROB of each included study. Any disagreements that cannot be resolved will be discussed with a third party (QZ). We will assess the ROB for each study following the Cochrane Collaboration approach for both randomized and non-randomized studies.^[27,28]^ The methodological quality and ROB of any randomized controlled trials will be qualified using the Cochrane Collaboration's tool for ROB (ROB) assessment.^[29]^ This tool assesses ROB include reporting bias, selection bias, detection bias, performance bias, and attrition bias. Each domain ROB will be assigned a ROB category as “low”, “high”, or “unclear”. The Critical Appraisal Checklist for Analytical Cross Sectional Studies from The Joanna Briggs Institute will be applied to assess the quality of cross-sectional studies.^[30]^ This tool consists of 8 items that could be scored as “yes”, “no”, “unclear” or “not applicable”. The 9-point Newcastle-Ottawa Quality Assessment Scale^[31]^ will be used to assess the quality of longitudinal studies, including case-control, and cohort studies. This tool includes 8 items grouped into 3 categories: selection, comparability, and exposure (case-control studies) / outcome (cohort studies). The NOS score ≥ 7 will be considered as high-quality. A summary ROB table will be produced, with an additional table briefly justifying each judgement included in the appendix. Publication bias will be assessed graphically by funnel plots. If the funnel plots show asymmetry, we will apply the Egger regression test.^[32]^ We will use RevMan 5.3 to pool the data for analysis.

### 2.5 Statistical analysis

#### 2.5.1 Assessment of heterogeneity

The choice of whether to conduct a meta-analysis and which model to use (fixed or random effects) will depend on the level of statistical heterogeneity assessed by the I^2^ index. An I^2^ value less than 50% represents a non-substantial level of heterogeneity. Substantial heterogeneity was considered where I^2^ was > 50%. If there is no evidence of heterogeneity, a fixed effect model^[33]^ will be adopted for the meta-analysis; otherwise, a random-effects model^[34]^ will be used.

#### 2.5.2 Data synthesis and analysis

If there are sufficient data in the selected studies with the same design and sufficiently homogeneous populations, exposures, and outcomes to calculate pooled effect estimates, we will consider performing a meta-analysis. If meta-analysis is not feasible due to high heterogeneity of studies, we will conduct a narrative synthesis. We will summarize the evidence for the association between COVID-19 and changes in the intestinal flora.

#### 2.5.3 Subgroup and sensitivity analyses

If substantial heterogeneity detected, subgroup analyses, and meta-regression will be conducted to look for the potential causes. If sufficient data is collected, a priori variables of interest for subgroup analyses to explore statistical heterogeneity will include:

(1) type of study design;
(2) country;
(3) characteristics of population;
(4) underlying disease or comorbidities;
(5) sample size;
(6) covariates included in the original studies (age, sex, weight, height, body mass index, among others);
(7) medication usage (antiviral therapy or intestinal management). Sensitivity analysis will be conducted to assess the robustness of summary estimates by removing the study one by one.

### 2.6 Ethics and dissemination

Ethical approval is not required for this study, as it is a systematic review. The results will be disseminated by publication of the manuscript in a peer-reviewed journal and for national and international presentations.

## 3 Summary

To date, no review has comprehensively explored the association between COVID-19 and intestinal flora. The protocol will facilitate an understanding of the impact of COVID-19 on intestinal flora. An increased understanding of the relationship between COVID-19 and intestinal flora may be possible to inform methods of therapy or prevention. Given the high mortality and the public health burden, the conduct of the present systematic review is of high clinical, and practical relevance. The study will have broad representativeness by including individuals of all age groups and from all continents. However, potential limitations are inherent in conducting systematic reviews and meta-analyses. There may be poor method quality, publication bias, information bias, etc. Some strategies will be adopted to ensure the lack of ROB. Two independent reviewers will conduct the systematic review and meta-analysis, and a third researcher will be consulted when consensus is not reached or inconsistencies exist in data collection. Furthermore, existing guidelines, the meta-analysis of Observational Studies in Epidemiology statement, Preferred Reporting Items for Systematic Reviews and Meta-Analyses, and Cochrane Collaboration Handbook recommendations will be followed.

## Author contributions

The study concept was developed by FYL, XYL, and QZ. The manuscript of the protocol was drafted by FYL and critically revised by XXW and LM. HL developed and provided feedback for all sections of the review protocol and approved the final manuscript. The search strategy was developed by FYL and XXW. Study selection will be performed by XXW and QZ. Data extraction and quality assessment will be performed by FYL and QZ, with XYL as a third party in case of disagreements. All authors have approved the final version of the manuscript.

**Investigation:** Xinxin Wang, Qi Zhang.

**Methodology:** Xinyun Li.

**Supervision:** Ling Mi.

**Writing – original draft:** Fangyuan Li.

**Writing – review & editing:** Lu Hua.

## Supplementary Material

Supplemental Digital Content
