# Supplementary material for: The impact of COVID-19 on intestinal flora: A protocol for systematic review and meta analysis
Source: Medicine (Baltimore). 2020 Sep 25;99(39):e22273. doi: 10.1097/MD.0000000000022273 (PMC7523765; doi:10.1097/MD.0000000000022273)
Supplement: Supplemental Digital Content [file medi-99-e22273-s001.docx]

**Appendix 1. Searching strategy in PubMed**

***#1****: "COVID-19" [Supplementary Concept,Mesh]*

***#2****:"2019 novel coronavirus disease"[Title/Abstract] OR "COVID19"[Title/Abstract] OR "covid 19 pandemic"[Title/Abstract] OR "sars cov 2 infection"[Title/Abstract] OR "covid 19 virus disease"[Title/Abstract] OR "2019 novel coronavirus infection"[Title/Abstract] OR "2019 ncov infection"[Title/Abstract] OR "coronavirus disease 2019"[Title/Abstract] OR "coronavirus disease 19"[Title/Abstract] OR "2019 ncov disease"[Title/Abstract] OR "covid 19 virus infection"[Title/Abstract]*

***#3****:* *"Gastrointestinal Microbiome"[Mesh]*

***#4****:* *"Gastrointestinal Microbiomes"[Title/Abstract] OR "Microbiome, Gastrointestinal"[Title/Abstract] OR "Gut Microbiome"[Title/Abstract] OR "Gut Microbiomes"[Title/Abstract] OR "Microbiome, Gut"[Title/Abstract] OR "Gut Microflora"[Title/Abstract]) OR "Microflora, Gut"[Title/Abstract]) OR "Gut Microbiota"[Title/Abstract]) OR "Gut Microbiotas"[Title/Abstract]) OR "Microbiota, Gut"[Title/Abstract]) OR "Gastrointestinal Flora"[Title/Abstract]) OR "Flora, Gastrointestinal"[Title/Abstract]) OR "Gut Flora"[Title/Abstract]) OR "Flora, Gut"[Title/Abstract]) OR "Gastrointestinal Microbiota"[Title/Abstract]) OR "Gastrointestinal Microbiotas"[Title/Abstract]) OR "Microbiota, Gastrointestinal"[Title/Abstract]) OR "Gastrointestinal Microbial Community"[Title/Abstract]) OR "Gastrointestinal Microbial Communities"[Title/Abstract]) OR "Microbial Community, Gastrointestinal"[Title/Abstract]) OR "Gastrointestinal Microflora"[Title/Abstract] OR "Microflora, Gastrointestinal"[Title/Abstract] OR "Gastric Microbiome"[Title/Abstract] OR "Gastric Microbiomes"[Title/Abstract] OR "Microbiome, Gastric"[Title/Abstract] OR "Intestinal Microbiome"[Title/Abstract] OR "Intestinal Microbiomes"[Title/Abstract] OR "Microbiome, Intestinal"[Title/Abstract] OR "Intestinal Microbiota"[Title/Abstract] OR "Intestinal Microbiotas"[Title/Abstract] OR "Microbiota, Intestinal"[Title/Abstract] OR "Intestinal Microflora"[Title/Abstract] OR "Microflora, Intestinal"[Title/Abstract] OR "Intestinal Flora"[Title/Abstract] OR "Flora, Intestinal[Title/Abstract]" OR "Enteric Bacteria"[Title/Abstract] OR "Bacteria, Enteric"[Title/Abstract]*

***#5****: (#1 OR #2)* ***AND*** *(#3 OR #4)*
